# Development of an Optical Method for the Evaluation of Whole Blood Coagulation

**DOI:** 10.3390/bios11040113

**Published:** 2021-04-09

**Authors:** Marinos Louka, Efstathios Kaliviotis

**Affiliations:** Department of Mechanical Engineering and Materials Science and Engineering, Cyprus University of Technology, 3036 Limassol, Cyprus; ma.louka@edu.cut.ac.cy

**Keywords:** blood coagulation, image sensing, image classification

## Abstract

Blood coagulation is a defense mechanism, which is activated in case of blood loss, due to vessel damage, or other injury. Pathological cases arise from malfunctions of the blood coagulation mechanism, and rapid growth of clots results in partially or even fully blocked blood vessel. The aim of this work is to characterize blood coagulation, by analyzing the time-dependent structural properties of whole blood, using an inexpensive design and robust processing approaches. The methods used in this work include brightfield microscopy and image processing techniques, applied on finger-prick blood samples. The blood samples were produced and directly utilized in custom-made glass microchannels. Color images were captured via a microscopy-camera setup for a period of 35 min, utilizing three different magnifications. Statistical information was extracted directly from the color components and the binary conversions of the images. The main advantage in the current work lies on a Boolean classification approach utilized on the binary data, which enabled to identify the interchange between specific structural elements of blood, namely the red blood cells, the plasma and the clotted regions, as a result of the clotting process. Coagulation indices produced included a bulk coagulation index, a plasma-reduction based index and a clot formation index. The results produced with the inexpensive design and the low computational complexity in the current approach, show good agreement with the literature, and a great potential for a robust characterization of blood coagulation.

## 1. Introduction

Blood flow allows the distribution of oxygen and nutrients to the organs and tissues. The importance of blood circulation is obvious in cases where health related problems affect people’s daily life. For instance, the conditions of hemophilia and hyper-coagulation have a significant impact on patients mortality rate. Hemophilia can cause major internal and external uncontrollable bleeding from minor injuries, due to the inability of proper clot formation. On the other hand, hyper-coagulation is associated with abnormal and uncontrollable formation of blood clots in the absence of injuries, or the inability of fibrinolysis to control clot growth and clot dissolution. Both conditions cause pathological problems and patients in many cases need daily monitoring in order to regulate their medication [1].

Hemostasis is a physiological process in which blood loss due to vessel injury is minimized by clotting, so that blood flow is maintained normal in the vessels. The hemostasis process involves the mechanisms of clot formation at the site of the injury, the prevention of uncontrollable clot growth, and the lysis (dissolution) of the clot. This process is characterized by a series of reactions which involve platelets, red blood cells (RBCs), coagulation factors and anticoagulants in a three-stage procedure. In the first stage of the process, named the primary hemostasis, an initial soluble white clot is created, consisting mainly of activated platelets. Activated platelets, at the site of the endothelial injury, bind with Von Willebrand factor through the GPIb-receptor [2]. Von Willebrand is a glycoprotein produced in the endothelial cells and it is critical to the initial clotting as it helps the aggregation of the platelets at the site of the injury. In the next stage, the secondary hemostasis (also known as blood coagulation), the inactive coagulation factors, that exist in the plasma under physiological conditions, are activated. In a complex process the coagulation factors promote the transformation/conversion of the soluble white clot and fibrinogen into an insoluble, stable clot and fibrin mesh, that plugs the damaged vessel [3,4].

Blood coagulation is described by the coagulation Cascade (or Waterfall model), which includes the intrinsic and extrinsic pathways, both leading to the common pathway. The final stage of hemostasis regards the deactivation of the coagulation factors and the dissolution of the clot to prevent oversize and unnecessary clotting at the site of the injury, which can affect the blood flow. This stage is known as fibrinolysis and consist of natural inhibitors that terminate the growth of clots and break them down. All three of the aforementioned stages require proteins, coagulation factors and natural anticoagulants to initiate and complete hemostasis [5]. Imbalance between coagulation and anticoagulation proteins as well as deficiencies in any of them, can cause bleeding disorders like hemophilia A, hemophilia B and Von Willenbrand disease, or arterial/venous thrombosis [6,7,8].

When blood is removed from the body and placed in a glass test tube, red blood cells (RBCs) are able to aggregate due to low shear stress, blood clots fairly quickly, and the blood coagulation process can be assessed immediately. Red blood cell aggregation (RBCA) causes the formation of stacks of RBCs in coin-like structures (termed rouleaux), the formation of larger aggregates from rouleaux combination, and even a 3-dimensional network of aggregates [9]. The result of the contraction of RBCs to aggregates is the appearance of larger plasma regions in the blood sample. Calcium ions are required for the clotting process, whereas acidic substances like EDTA (ethylenediaminetetraacetic acid) or citrate can prevent the formation of clots by binding calcium. EDTA and citrate are used widely in the Hematology laboratory as anticoagulants. Clotting can be initiated in vitro at a later time by adding back an excess of calcium ions [10].

A variety of laboratory equipment and point-of-care (POC) devices have been developed through the years for blood coagulation assessment, exploiting a range of blood properties [11]. In those tests, platelet count, functionality, adhesion and aggregation are of primary importance. Platelet testing provides valuable information concerning patients with bleeding disorders; it allows to monitor patient’s medication treatment, assess in-surgery hemostasis and guide transfusion medicine with manual or automated instruments [12]. The cost of hand-held POC devices and their disposable strips is not negligible, whereas time and complexity might be an issue for the techniques (such as thromboelastography) that exploit the mechanical properties of blood coagulation.

The extrinsic and intrinsic pathways can be used for in vitro laboratory test screening, extracting parameters like the prothrombin time (PT) and activated partial thromboplastin time (aPTT), respectively [13]. PT and aPPT indicate the time that it takes for clots to form in plasma when calcium and tissue thromboplastin are present, and their magnitudes depend on device specific parameters. The extrinsic pathway test, aPTT, is sensitive to factors, I, II, V, VII, X, whereas the intrinsic pathway test, PT, is sensitive to factors XII, XI, IX, VIII, and further, sensitive to deficiencies of prekallikrein and high molecular weight kininogen (HMWK). These indices can be derived with a variety of instruments, with each of the instrument exploiting different blood coagulation properties [14]. Also, an important widely used index for blood coagulation is the International Normalize Ratio (*INR*) which is derived from the PT test and it is used for effective monitoring patients under oral anticoagulation therapy [15]. *INR* is calculated by the formula:

(1) $$\;INR={\left(\frac{PT_{patient}}{MNPT_{patient}}\right)}^{ISI}$$

where $MNPT_{patient}$ is the mean normal *PT* of a control group and ISI is the International Sensitivity Index, which is used to adjust sensitivity difference of a variety of reagents used by instruments.

Electrochemical techniques for analysing coagulation are widely adopted in Hematology laboratories and POC devices. These techniques are based on resistance/capacitance changes of whole blood as clot related macromolecules react to the added agents in the sample. During the coagulation process the dominant impedance is moved from the plasma impedance to the aggregated red blood cells, and finally to the fibrin network impedance [16]. As blood coagulation escalates in time, the total impedance increases and can be accurately measured and provide the *INR* index, using the formula in Equation (1). This method leads to smaller and patient friendly POC devices as it is based on integrated electronics platforms and disposable test strips, with examples like ${\mathrm{CoaguChek}}^{®}$ XS (Roche Diagnostics, Indianapolis, IN, USA), microINR (${\mathrm{iLine}}^{®}$ Microsystems S.L., San Sebastian, Spain) and i-STAT (Abbott Point of Care Inc., Abbott Park, IL, USA). However as mentioned, the cost of such devices and their disposable strips is not negligible [17,18].

Another approach to assess further details of the blood coagulation process is the Thromboelastography method ($TEG^{®}$, Haemonetics, Braintree, MA, USA), which examines the mechanical characteristics of blood in terms of viscoelasticity and kinetics as blood clots form. TEG was first introduced by Hertert in 1948 [19], as a global essay that characterizes in real time the creation of clots in whole blood using a container for the sample and a constant forced rotating rod immersed into the sample. The rod is connected to a transducer, which measures the torque generated by the developed clot. Rotational thromboelastometry ($ROTEM^{®}$ TEM International, Munich, Germany) is considered the evolution of TEG, following the same fundamental principles. Both exploit the viscoelasticity on whole blood as coagulation and lysis occurs with the use of reagents, producing essentially similar indices based on characteristics of the generated curves. The basic parameters in TEG and ROTEM need 30 to 60 min and are calculated in terms of the time-evolved curve amplitude (in mm) which indicates the clot firmness. Coagulation indices are derived from linear combination of a variety of the parameters like the maximum reached amplitude, kinetic time, clotting initiation time and the angle between the initiation of coagulation tangent to the developed curve [20]. It is also suggested that Thromboelastography can potentially help to guide patients needs for transfusions in surgery and trauma induced coagulopathies, through specialized algorithms embedded in the devices [21,22].

Coagulation in blood causes intense structural changes that can be detected by image analysis techniques. Machine vision approaches offer a range of methods and techniques for image processing [23], and are used for the development of Lap-on-Chip devices [24]. These techniques can be used for real-time monitoring and characterization, in biomedical research and clinical diagnostics [24]. Methods such as image segmentation allow the identification of structures with specific characteristics [25,26]. To accommodate the blood sample to be processed, simple microfluidic configurations are often used, offering several advantages [27,28,29,30]. Other techniques, such piezocoagulography, have been also utilized for blood coagulation, showing also the possibility of studying the initial stage of fibrin formation in the clotting process [31].

In recent works presented in the literature, the optical properties of coagulating blood are exploited, by measuring the light propagation and/or scattering through a sample (please see [11] for a review on the subject). The produced time-varying curves provide valuable information about the blood coagulation process. This technique, termed photometry, requires the acquisition of sequential light patterns, usually images, utilising a light source and a camera. Photometric results can be achieved with monochromatic or polychromatic light sources, and experimental setups covering the infrared to the x-ray spectrum, utilizing appropriate cameras. It has been shown that light sources with a wavelength around 690 nm (near-infrared window) provide very good results, due to the minimal light absorption by hemoglobin and water [32]. During coagulation, the light patterns acquired by the camera change dynamically as clots develop in the sample. Furthermore, light transmission through the sample is blocked by the newly formed clots and the averaged detected light is decrease due to the interaction between the platelets, the RBCs and the procoagulant plasma proteins [33,34]. The aforementioned phenomena can be characterized, after digitally process the captured images. Several studies in the literature utilize laser speckle analysis between consecutive images, and/or between a reference image (usually the initial one), in combination with cross- or auto-correlation techniques, which can be computationally demanding. The results are presented as time-varying curves with the potential of dividing them into different coagulation phases [35,36,37]. Indices extracted from those curves can describe blood coagulation and typically are validated by comparison with commercially available coagulometers [38,39]. Techniques, such us the aforementioned, utilize color spectra to assess blood coagulation and extract useful indices, by using a specific light wavelength. The produced information can be visualized by using pseudo-colored maps and as a result, they may lack a direct representation of the whole blood clotting process. Moreover, such techniques require specialized components and set-ups, such as laser diodes, optical components (splitters, expanders, polarizers, etc.).

In this work, an optical assessment technique for blood coagulation is presented, using whole blood from a healthy volunteer, with conventional bright-field microscopy. The tests were conducted using capillary whole blood obtained by finger prick without added reagents. Image processing algorithms have been developed to analyze a series of images acquired from a camera/microscope setup, in gray, color and binary levels, using three magnification lenses and custom-made microchannels. The produced curves display three distinct phases, the RBCA, the clot development and the completion of whole blood coagulation. A classification algorithm separates the images, by detecting the areas in which clots are developed. Results extracted from the four color channels (red, green, blue and gray scale) show good sensitivity and conclude that this method can be used to assess whole blood coagulation with low computational demands and inexpensive equipment. The current work is set in order to demonstrate the validity and the potential of the proposed approach.

## 2. Materials and Methods

### 2.1. Sample Preparation

The blood donation protocol for this work was approved by the Cyprus National Bioethics Committee (permission ref.: EEBK/EP/2019/19). A drop of blood was collected through a finger prick from a healthy volunteer (following 10 h of fasting) after the application of intermittent pressure to a finger in order to increase the blood flow. Then the area was cleaned with alcohol and a sterilized lancet was used to puncture the side of the fingertip. The first drop of blood was wiped with a sterilize cotton ball and the second drop of blood was placed at the entrance of a custom-made microchannel. The blood drop was then drawn by capillary forces in the observation area. A total of three different experiments were made and presented in this work, at three different microscope objectives. Each set of tests was completed in approximately two hours.

### 2.2. Experimental Setup

**Figure 1:**
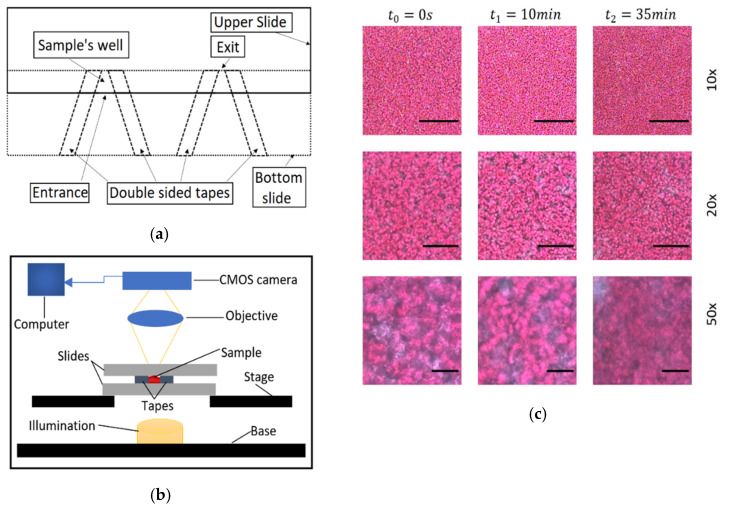
(**a**) The design of the microchannels. (**b**) Schematic of the experimental setup, which is based on forward propagation of light into to the sample, through the objective and into a CMOS camera for the acquisition of data. (**c**) Representative images showing the initial, an intermediate and a final state of coagulated blood at the 10×, 20× and 50× magnification objectives. Scale bars equal to 200, 100 and 40 μm for the 10× 20× and 50× objectives, respectively.

The microchannels were composed by two microscope glass slides (75 mm × 25 mm × 1 mm, for length, width and height, respectively) and double-faced adhesive tapes (${\mathrm{Tesa}}^{®})$ with dimensions 12.7 mm width and approximately 100 μm thickness. The tape was adhered in pairs and at a small angle between them, on one slide (Figure 1), creating an open space. The second glass slide was placed above the first one and adhered on the tapes, creating the microchannel and effectively the testing site. Two testing sites were created on the same glass slide. The glass slides overlapped for approximately 5 mm length, with entrance and exit widths of approximately 5 and 3 mm respectively, resulting in a small microchannel volume of approximately $2\;{\mathrm{mm}}^{3}$. The angle of the tapes and the relatively small distance from the entrance to the exit resulted in fast filling, and fast deceleration and immobilization of the blood in the channel. After the placement of the sample in the channel, sunflower oil was applied at the entrance of the microchannel to isolate the sample from air, and restrict undesirable effects, such as air bubble formation and blood movement. The viewing window was at the center of the geometry at distances of approximately 2 mm from the entrance and exit. This region of interest in the center of the geometry, appeared to have uniform characteristics, not influenced by the boundaries of the channel.

For video/image acquisition a JVC TK-C1380 camera was used, integrated on an Olympus BX51 microscope (Figure 1). The microscope light illumination (100 W long-life halogen bulb) was controlled by an intensity knob which was calibrated and scaled utilising an adhered protractor around the knob. Tests were conducted to validate the linearity between the detected light intensity and the protractor’s degrees, with a deviation of around ±5 degrees. So, the protractor was utilized to set the light intensity appropriately, with a fine approximation in degrees. Before sampling, the camera focus was optimized manually by adjusting the microscope stage, and by observing the camera image to avoid opaqueness and loss of detail.

The camera was connected to a computer in which the video was saved. Three experiments were conducted with different magnifications lenses of 10×, 20× and 50× of numerical apertures of 0.25, 0.40 and 0.50, respectively, using blood sample from the same volunteer. Each acquired video has a 35 min duration using the free software VirtualDub at 5 frames per second (fps), enough to detect the blood coagulation process. Representative images from the three magnifications and at different times are shown in Figure 1. The resolution of the camera/microscopy setup was 1.0 μm/pixel, 0.55 μm /pixel and 0.22 μm/pixel ±10% for the 10×, 20× and 50× respectively, the observation image area was 576 × 768 pixels, and the processed image area were 500 × 500 pixels.

### 2.3. Whole Blood Sample Coagulation Evaluation

Blood coagulation was evaluated by digitally processing the acquired videos utilizing the developed algorithms (The MathWorks Inc., Matlab-Natick, MA, USA). The video acquisition rate was initially set to 5 fps, however various reduced sampling rates were used in the processing stage in order to minimize storage needs and optimize the performance in terms of overall processing time. Apart from various sampling rates, different image sizes were used for performance optimization in the processing stage, while keeping the main body of the algorithm unchanged. After the evaluation of the produced time-depended indices, the minimum frame rate, preserving the details of the process, was found to be at 4 s per frame (0.25 fps), with total number of images per experiment to be 525 colored images.

The processing stage can be divided into three main sections, with each one calculating various values and parameters of interest, from the time-varying image series. First, each image is separated into their red, green, blue and gray-scale (RGBGs) color channels. For each of the four color channels the mean intensity (MI) value is calculated for each image and for all sequential images from the various tests. The MI corresponds to the average light travel through the sample at each specific moment. MI curves for all color channels were smoothed in a moving average manner in order to extract more meaningful information at the initial parts of the process (up to approximately 10 s), where the data appear noisier. The data were further normalized with the maximum values of the time series (and denoted as $MI^{\;*}$), so that their time response of the various cases can be directly comparable.

In the second algorithmic section, the images were converted into binary data (i.e., black and white pixels), using a unique threshold for each color channel, and for each experiment. In the initial stages of the process, when no clots are present, the binary images have pixels with values one (white pixel) and zero (black pixel), representing the plasma and RBCs areas, respectively. This is due to the fact that the transmission of light through the plasma is higher than the transmission of light through the RBCs and clots (see for example the sample image at $t_{0}$, and at 50× magnification in Figure 1). So, as RBCs block more light than the plasma, their gray scale values is lower to that of the plasma pixels. In the transformation of a gray scale image into binary the threshold value is the most important parameter. The threshold is a gray scale value that separates all the pixels into two categories, those above and those below the threshold value. The RGBG thresholds were based on the image characteristics in the peak value of their $MI{\;}^{*}\;$ curve. That threshold value was selected for two reasons. First, at that point in time the phenomenon of RBCA is mostly complete, as it takes around three-four minutes to fully develop, and blood coagulation is the main function that affects the structure of the blood sample. Until this point an increase in the averaged light sensing was observed. Thus, this time point can be referred to as the clotting start (CS) time after which blood coagulation can be studied. Secondly, by deriving the binarization threshold value from the CS correspond image (resulting in a high threshold value), ensures the efficient detection of the growing clots in the subsequent analysis, as the distribution of pixel intensity will lean towards lower values due to blood coagulation. As the new structures, the clots, are forming they eventually assigned zero pixel values, due to their light-blocking (absorbing) nature. Thus, the area occupied by clots increase in size, by the action of coagulation factors that blood plasma contains, and the corresponding plasma area is decreased. As a result, the percentage of the white pixels correspond to the plasma area is decreased in time. Therefore, the black pixels with zero values after processing, represent RBCs (and their aggregates) and the newly formed clots. A significant advantage of the present algorithm is that RBCs and newly formed clot areas can be distinguished and properly analyzed.

In the third section of the algorithm a Boolean algebra calculation loop was developed to extract indices from the binary image series with reference to the image at the CS time, in a pixel by pixel manner, similarly to methods used in studying elastic optical networks [40]. Karnaugh mapping (KM) was used to simplify the expressions using logical operators. As mentioned above, the clot detection was classified as the change of pixels with values one to zero (i.e., from plasma pixel to clots pixel). As each binary image only consists of two values, ones and zeros, there are four possible combinations when comparing two images, each corresponding to a specific condition/stage regarding the blood coagulation process. The first combination is the zero to zero pixel value that regards the detection of RBCs or RBCA region that remains unchanged. The second regards changes from a zero value to one, denoting a new plasma area (NPA) which can appear due to the RBC aggregation (RBCA), in which RBCs attract each other leaving an empty from cells space (plasma). This procedure however is considered complete after the initial 4–5 min as mentioned above. In a third combination, a change from one to zero corresponds to the clot formation (CF) in areas where previously were occupied by plasma. In the fourth combination, values compared from one to one represent the plasma area (PA) still unoccupied by blood clots. As in the above section of the algorithm, the change of a white pixel to a black pixel is to be expected and indicates the activity of the coagulation process in the sample. For the four pixel comparisons mentioned above, the image corresponding to CS time is used as the reference image. Using the results of the pixel comparison the algorithm can separate areas as RBCs, the newly formed clots (CF), the initial plasma area (PA) at CS, and the new plasma areas (NPA).

Table 1 shows the truth table of the aforementioned combinations, and gives information about the four outputs, which can be conceived as four new images. Each new image has binary pixel values according to the output.

$I{\left(\tau \right)}_{i,j}$ denotes the value of the pixel in the ith row, jth column of the reference image and $I{\left(\tau +t\right)}_{i,j}$ the corresponding pixel of the sequential image at a time τ+t, where τ is the CS time. The outputs RBCs, NPA, CF and PA are new binary data (images) at a given time, after CS, and they have the same size as the compared images, representing specific structural conditions in blood. The information from the Table 1 and the utilization of KM are used to derive indices describing, and quantifying, each of the aforementioned conditions in every image. The indices are developed according to Equations (2)–(5), with N noting the number of pixels of the image (total number of columns $N_{i}$ and total number of rows $N_{j}$ are equal to N, $\bar{I}$ denoting the complement pixel value of I at that position (logical NOT operation), and with the symbol ^ denoting the logical AND operation. For each combination and for all images, the total number of ones is divided by the total number of pixels in the image, resulting in the expression of the indices as fractional quantities (denoted with the * superscript).

(2) RBCs *= 1N∑i=1N∑j=1NI¯(τ)i,j^I¯(τ+t)i,j

(3)  NPA *= 1N∑i=1N∑j=1NI¯(τ)i,j^I(τ+t)i,j

(4)   CF*= 1N∑i=1N∑j=1NI(τ)i,j^I¯(τ+t)i,j

(5)  PA*= 1N∑i=1N∑j=1NI(τ)i,j^I(τ+t)i,j

**Figure 2:**
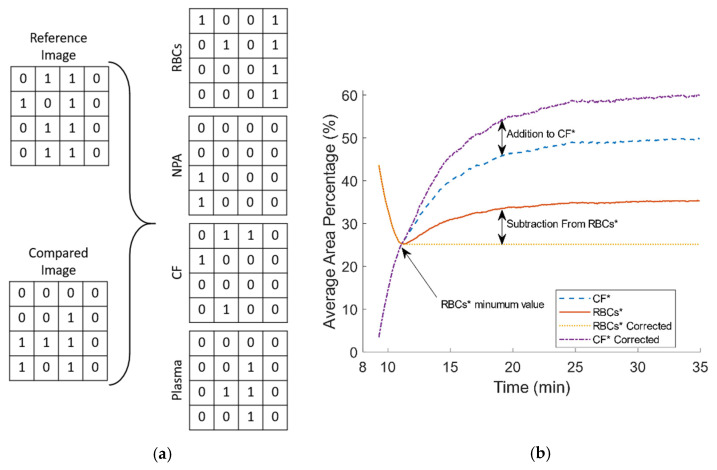
(**a**) Representative binary comparison between parts of a reference and a subsequent image, and the resulted output matrices. (**b**) The behavior of $RBCs{\;}^{*}$ and $CF{\;}^{*}$ after clotting start (CS) and the correction process.

The addition of Equations (3) and (5) represents the total apparent blood plasma at any given time, after the CS time. Moreover, one can see that the pairs Equations (2) and (5), and Equations (3) and (4) are complementary due to their definition from Table 1. Therefore, it is of interest to further analyse the relation between $RBCs^{\;*}$ and $CF{\;}^{*}$. Figure 2a shows sections from a reference and a compared image, and the four output matrices. Figure 2b illustrates the behavior of $RBCs{\;}^{*}$ and $CF{\;}^{*}$. As mentioned earlier, the RBCA process was mostly complete at CS, as normally reaches a steady state after around 120 s. However, this time period could be extended due to various reasons. For example, small cell movements inside the microchannel could contribute to a prolonged RBCA time. After CS, a small activity of RBCA was observed for a small period of time, and that resulted in a small increase in $NPA^{\;*}$ and a small decrease in $CF{\;}^{*}$. The apparent increase of $RBCs{\;}^{*}$ after it has reached a minimum (which denotes the completion of RBCA) seems artificial, since it is caused by the re-appearance of a zero value pixel area that should correspond to $CF{\;}^{*}$, at the same location where previous $NPA^{\;*}$ was detected. In terms of pixel values, a zero in the reference image had initially compared with one (corresponding pixel in the subsequent image) but at a later time it was compared with a zero again. However, this condition most probably corresponds to the detection of clots in the region of NPA and not to a new RBCs. In a correction process, the increase that occur from the re-appearance of $RBCs{\;}^{*}$ is subtracted, and the subsequent increase is added to $CF{\;}^{*}$ as Figure 2b shows.

In order to distinguish the most efficient image resolution and color channel for the image processing, a sensitivity index (SI) was developed (Equation (6)) to compare the resulted curves. The SI was applied only to the plasma reduction (*PR*) and the CF curves, as the $MI{\;}^{*}$ curves are normalized using their own limits. *PR* is defined as the ratio of the total plasma area to the total image area (Equation (7)). SI takes in consideration the upper and lower limits (maximum and lower PR values) and calculates the percentage of change in that time interval. Evidently, the higher the value of SI the more susceptible is a color channel to change. SI follows the formula:

(6) $$\;SI=\;\frac{\left|\min(V_{ch})-\max(V_{ch})\;\right|}{\max(V_{ch})\;}\times 100$$

$V_{ch}$ is the value of a color channel at a specific objective magnification, and at a specific point of interest (maximum or the minimum value).

**Figure 3:**
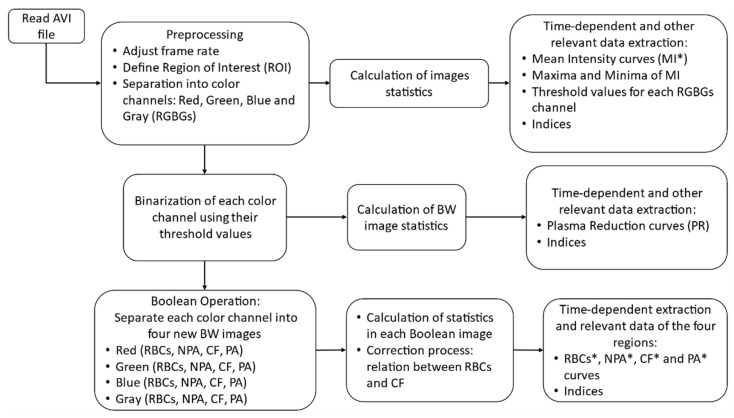
Flowchart of the proposed algorithms showing the three stages.

Figure 3 shows a flowchart representing an overview of the algorithm. Initially the input data, an AVI file, is preprocessed in three steps: adjustment of the frame rate (reduction needed fps), define and crop the region of interest, and separate the resulted colored images into their red, green, blue and gray components. Then, the first statistical analysis is performed to produce relevant indices and other parameters (i.e., threshold values for binarization, and normalization). At the second part (second row in the flowchart) the previously processed images are converted into binary, and various statistics are calculated. Finally, in the last part of the algorithm (last row of the flowchart), Equations (2)–(5) are implemented to derive the four new regions in each binary channel and to calculate the relevant statistics and indices.

**Figure 4:**
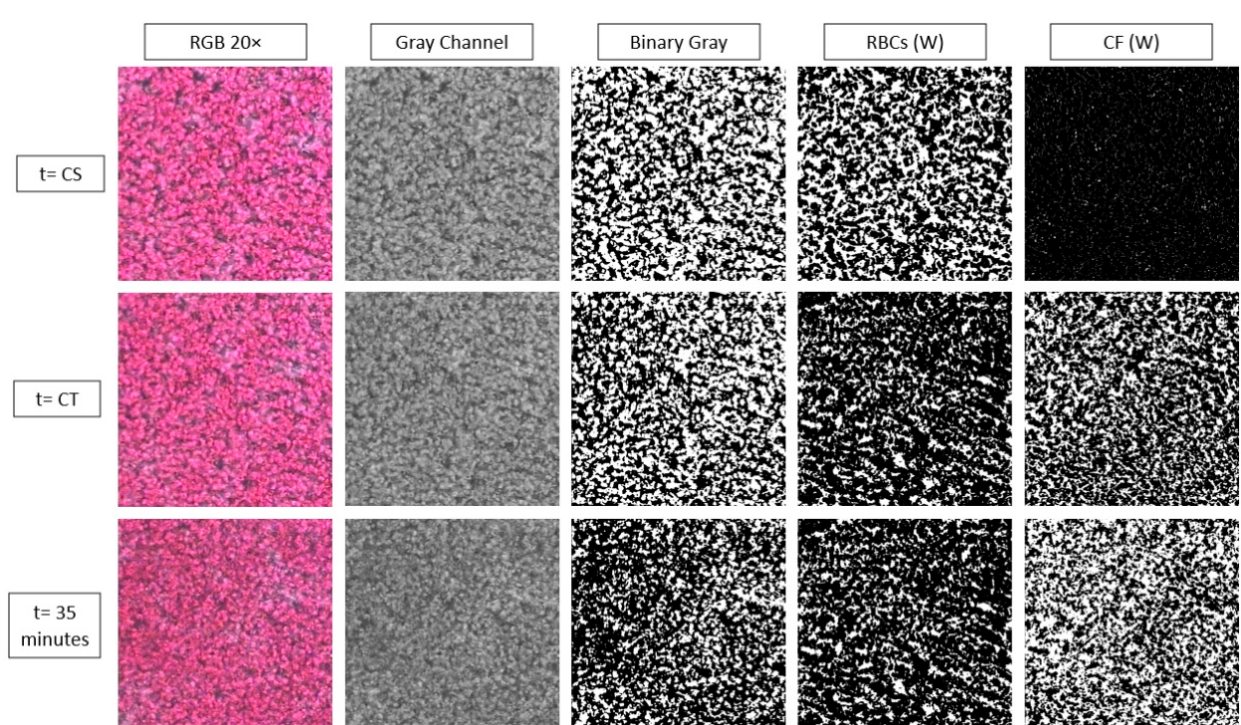
Representative images and the resulted red blood cell (RBC) and clot areas (CF) in white (W) pixels, on which the relevant indices are defined.

Figure 4 below shows the result of the processing algorithm for the 20× objective and for the gray level images, as the representative of the cases. As it can be seen the clot formation is detected as negligible in the initial time (*t* = CS), following a substantial increase at the half time of the coagulation process (*t* = CT), and reaching a maximum at the end of the time (*t* = 35 min). The area occupied by the RBCs is decreasing in time as the clotted regions extent on the sample. As noted earlier the plasma area (not shown) is complementary to the RBC area at the early stages, as coagulation progresses however, the plasma and RBC areas are occupied by clots, resulting in the CF images.

## 3. Results and Discussion

**Figure 5:**
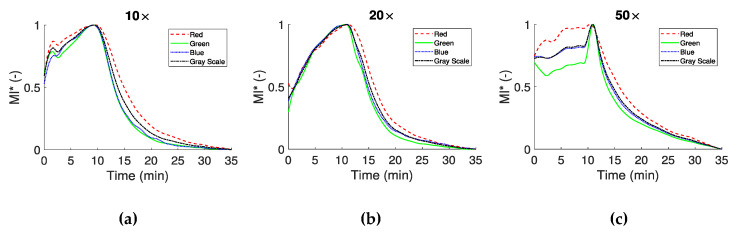
Normalized mean intensity $MI{\;}^{*}$ of the RGBGs responses for the three different magnifications (10× in panel (**a**), 20× in panel (**b**) and 50× in panel (**c**)). The maximum value of MI is used for the normalization.

Three approaches have been utilized in this work for the whole blood coagulation assessment. In the first approach the mean intensity (MI) for each color channel was calculated for the three different magnification objectives. The images were separated into their four color channels in order to detect differences between them, regarding blood coagulation. The mean image intensity was examined in order to assess the overall effect of light blocking and the characteristics of whole blood coagulation time. The MI index for all color channels in Figure 5 has been normalized with its maximum value (and denoted as $MI^{\;*}$) in order to compare the behaviour of the different color channels. In this figure the time response of the RGBGs channels at three different magnifications is illustrated for the whole experimental period. In all cases, there is an increase in the average detected light until approximately the 10th minute, most probably due to the phenomenon of RBCA, in combination with the initial stages of fibrin formation [31]. After RBCA reaches a maximum and any cell movement has ceased, blood coagulation is the dominant process affecting blood microstructure. Indeed, after approximately 10 min from the peak of $MI^{\;*}$, there is a significant decrease in the MI curves as light is blocked due to the structural changes in the sample, caused by the formed clots.

$MI^{\;*}$ curves appear in the same form for the specific magnifications, with some differences between the color channels. Differences are observed for the initial parts of the curves, however it should be noted that for each magnification a different test was performed. For example, in Figure 5a a spike like behavior around the second minute is observed, most likely due to a movement of blood components. In Figure 5c for the 50× objective, an obvious difference exists at the beginning of the process between the red and green color channels. In this magnification, small displacement of blood in the microchannel may have a greater effect in the results. After the maximum of the curves (which define the CS time), all curves have a similar behavior, with small differences between the experiments (i.e., the three different magnifications) and the color channels. The CS times observed in the present work for the non-reacted normal blood sample are in the vicinity ~10 min, agreeing with times observed in other studies. For reacted blood samples the onset of the coagulation process is detected at much smaller times depending on the reaction agent [16,35,36,38].

In the second approach the images were converted to binary, as described earlier. The binarization of the data resulted in the separation of the image into regions of plasma, RBCs and clots, and subsequently to the definition of the $RBCs^{\;*}$, $NPA^{\;*}$, $CF^{\;*}$ and the $PA^{\;*}$ indices. These indices were calculated for all consecutive frames from the CS time onward. As blood coagulates, and clots increase in number and size, the total number of pixels corresponding to plasma decrease could be assessed using the *PR* ratio:

(7) $$PR=\;\frac{Total\;Plasma\;Area}{Total\;number\;of\;pixels}$$

**Figure 6:**
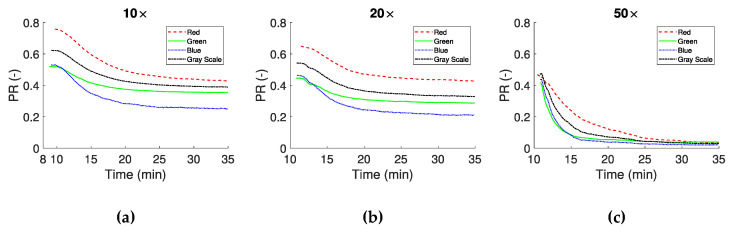
Plasma reduction (*PR*) as the ratio between the RBCs/clots and the plasma, of sequential images in time after CS. Panels (**a**–**c**) show the results from the 10×, 20× and 50× magnifications respectively.

*PR* is presented in Figure 6 for the 10×, 20× and 50× objectives, showing similar behavior between channels. This is expected, according to the $MI{\;}^{*}$ curves in Figure 5a,b, which have a similar behavior after CS. Figure 6c shows a greater reduction in the initial value of *PR*, probably due to a lower threshold value in the processing stage caused by the higher objective magnification.

**Figure 7:**
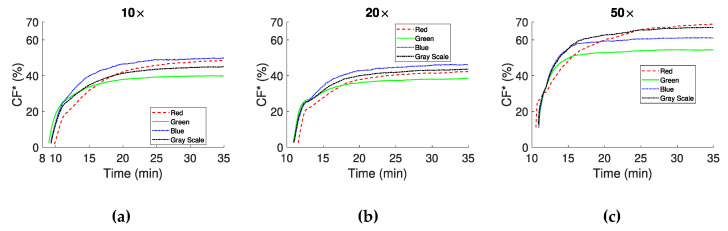
$CF{\;}^{*}$ expressed as percentage of the image area that is occupied by clots for the three magnifications (10× (**a**), 20× (**b**) and 50× (**c**)).

Information on clot formation, provided by the $CF{\;}^{*}$ index, is illustrated in Figure 7. Figure 7a,b correspond to 10× and 20× objectives respectively and show that $CF{\;}^{*}$ (clot area in the image) has similar time response and maximum values at the steady state. Differences are apparent regarding the maximum values between each color channel at steady state. In the 50× objective case (Figure 7c) the shape of the curves for the color channels remained similar to the other two objectives, however the maximum $CF{\;}^{*}$ values detected are higher. All $CF^{\;*}$ curves have an exponential characteristic, and show a rapid growth of the forming clots in the early minutes after CS.

One of the objectives of this study was to identify the most efficient approach in terms of blood coagulation assessment using brightfield microscopy. Therefore, the results for $MI{\;}^{*}$, *PR*, and $CF{\;}^{*}$ can be further analysed for sensitivity utilising the sensitivity index SI (Equation (6)). Table 2, Table 3 and Table 4 present the CS time, a characteristic coagulation time (CT), and SI for the three objectives used and for all color channels. CT is defined as the “half-life” time of the coagulation process after CS, i.e., the time required to reach the 50% of the $CF{\;}^{*}$ growth, or the 50% in $MI^{\;*}$ and *PR*, with maximum/minimum values defined at the 95% of the developed curves. The mean value and the standard deviation (SD) from the RGBGs channels are included for each index in the tables.

Inspecting Table 2, Table 3 and Table 4 is apparent that the Mean CS times in the $MI{\;}^{*}$ analysis show good agreement, with values of 9:18 ± 0:11, 10:54 ± 0:10 and 10:46 ± 0:14 min and seconds, for the 10×, 20× and 50× objectives, respectively. Similarly, for all objectives the CT of the $MI{\;}^{*}$ index shows similar Mean and SD values. Small differences in the Mean CS and CT values between the objectives could be attributed to the manual handling of the sample placement, and the sampling initiation time of the experiments. SD of the metrics CS, CT of $MI{\;}^{*}$, and CT of *PR* are very close to each other for all the color channels in the individual experiments.

Despite the similarities in the general behavior of the indices, minor discrepancies are also apparent. For example, for the 10× and 20× objectives the Mean and SD of the SI for the *PR* index are −38.44 ± 9.54% and 37.93 ± 9.87%, respectively. For the 50× objective, however, this metric is equal to −92.85 ± 1.96%, a much higher mean value with smaller SD than the other objectives. This might be due to the higher magnification in the 50× lens, where there is a smaller sampling area, and therefore small cell movements, due to RBCA and/or the formation of new clots, have a greater effect in allowing/blocking the transmitted light. In the $CF{\;}^{*}$ analysis, the difference in the SI values between the 10× and 20× with the 50× objective is smaller than previously observed, with Mean and SD values of 43.34 ± 4.22%, 39.64 ± 3.20% and 51.07 ± 6.44% for the 10×, 20× and 50× objectives, respectively.

The results of the present study are comparable with those from recent and earlier works exploiting the optical properties of blood coagulation. These works include the laser speckle contrast imaging (LSCI) methods [41], which are also non-contact techniques. LSCI methods quantify light scattering and propagation, using wavelengths in the infrared/near infrared light spectrum [36,37,38]. These studies have established an exponential behavior of the assessed coagulation indices, with characteristics depending on reagent and sample used. This exponential behavior is observed in the present study for the non-activated coagulation of blood in Figure 7a–c. Moreover, the present results agree both qualitatively and quantitatively with data obtained using TEG instruments, in which CS times of approximately 12 min and CT of approximately 4 min are observed [42].

A popular method of choice, in several of the aforementioned and other studies in the literature, for the extraction of plasma, or whole blood coagulation parameters, is the calculation of the (cross- or/and auto-) correlation coefficients in consecutive images. Correlation methods can quantify the similarities between two signals (images), however with a computational cost related to the number of arithmetic operations, usually scaling with the square of the sample number (image size and number of images). The total number of operations in the binary comparison used in the present study, scales directly with the sample number. The binary method utilized in this work results into specific regions of interest, depending on the desired complexity (four regions in the present case). The separation of the four regions requires a small amount of processing power due to low algorithmic complexity, as shown in the Equations (2)–(5). Also, the visible light source offers true color images that can be used to further analyze whole blood coagulation process using for example feature extraction algorithms.

## 4. Limitations and Sources of Uncertainty

The blood sample used in the study was provided by the same donor, therefore negligible differences in the sample are expected between the various tests. The repeatability of the technique is still to be addressed in detail, however, an indication for the robustness of the technique is given by the similar behavior of the indices, which have been obtained from different tests in each magnification. As mentioned earlier, some uncertainty is expected in the CS and CT times, due to sample handling and manipulation as the sample placement was performed manually. Environmental factors (e.g., room temperature and illumination, etc.) are not expected to have a significant effect, as they were controlled and consistent throughout the experiments. The in-house construction of the microchannels may have resulted in small geometrical differences, however no significant influence is expected in that aspect. Accurate focusing of the microscope on the sample may be an issue for the high magnification cases (50× objective), however by inspection no significant issues were identified.

In terms of image processing, the opaqueness of the sample and the probable higher concentration RBC stacks from the RBCA process, can decrease the overall light absorption by the image sensor. In the absence of the standard test, these results can be compared with data from literature [34,38]. Nevertheless, the efficiency of the image processing method adopted in this work is reflected in the analysis of the results and more specifically in the SI data.

## 5. Conclusions

In the present study, indices for the assessment of coagulation in a drop of blood were developed, and experiments were conducted in custom made microchannels, in order to demonstrate the validity and the potential of the approach. The different magnification lenses used showed major similarities and minor differences, regarding the behavior of the various indices. The coagulation process in whole blood was found to have an exponential behavior in agreement with other studies in the literature. The developed methodology presented in this work is very promising for application in small scale diagnostic devices for the point-of-care. Specifically, the current setup (brightfield illumination/camera imaging/low magnification microscopy/simple channel) is ideal for the construction of inexpensive POC devices. Further, the direct statistical analysis of the images, in combination with the Boolean approach can result in reduced computational needs, therefore allowing for onboard processing in a POC device.

## Figures and Tables

**Table 1:** The four combinations of the pixel-wise comparison between the image I at reference time τ (denoted as $I{\left(\tau \right)}_{i,j}$, and the subsequent images $I{\left(\tau +t\right)}_{i,j}$.

Inputs		Outputs			
$I{\left(\tau \right)}_{i,j}$	$I{\left(\tau +t\right)}_{i,j}$	$RBCs_{i,j}$	$NPA_{i,j}$	$CF_{i,j}$	$PA_{i,j}$
0	0	1	0	0	0
0	1	0	1	0	0
1	0	0	0	1	0
1	1	0	0	0	1

**Table 2:** Clot start time (CS), characteristic coagulation time (CT) and sensitivity index (SI) for mean intensity ($MI^{\;*}$), plasma reduction (*PR*) and clot formation ($CF{\;}^{*}$) in the 10 times objective case.

10×	*MI* *		*PR*		*CF*	
	CS(m:s)	CT(m:s)	CT(m:s)	SI(m:s)	CT(m:s)	SI(m:s)
Red	9:33	5:20	5:03	−40.54%	3:28	46.78%
Green	9:16	3:45	4:28	−28.20%	1:24	37.54%
Blue	9:05	4:00	4:20	−50.59%	2:12	46.15%
Gray	9:16	4:29	4:52	−34.41%	1:56	42.87%
Mean	9:18	4:24	4:41	−38.44%	2:15	43.34%
SD	±0:11	±0:42	±0:28	±9.54%	±0:52	±4.22%

**Table 3:** CS, CT and SI for mean intensity MI*, plasma reduction *PR* and clot formation $CF{\;}^{*}$ in the 20.

20×	*MI* *		*PR*		*CF*	
	CS(m:s)	CT(m:s)	CT(m:s)	SI(m:s)	CT(m:s)	SI(m:s)
Red	11:05	5:00	4:23	−30.71%	1:43	39.79%
Green	10:41	3:53	3:43	−32.43%	0:51	35.17%
Blue	10:57	3:53	3:47	−52.33%	1:35	42.67%
Gray	10:53	4:20	4:19	−36.26%	1:19	40.93%
Mean	10:54	4:16	4:03	−37.93%	1:22	39.64%
SD	±0:10	±0:32	±0:20	±9.87%	±0:22	±3.20%

**Table 4:** CS, CT and SI for mean intensity MI*, plasma reduction *PR* and clot formation $CF^{\;*}$ in the 50.

50×	*MI* *		*PR*		*CF*	
	CS(m:s)	CT(m:s)	CT(m:s)	SI (m:s)	CT(m:s)	SI(m:s)
Red	10:25	5:25	4:15	−92.45%	2:48	57.60%
Green	10:57	2:40	1:19	−90.36%	1:03	42.53%
Blue	10:53	3:24	1:35	−95.03%	1:11	50.20%
Gray	10:53	3:45	2:12	−93.57%	1:39	53.94%
Mean	10:46	3:48	2:20	−92.85%	1:40	51.07%
SD	±0:14	±1:09	±1:19	±1.96%	±0:47	±6.44%
